# Vitamin D3 inhibits micro RNA-17-92 to promote specific immunotherapy in allergic rhinitis

**DOI:** 10.1038/s41598-017-00431-1

**Published:** 2017-04-03

**Authors:** Zhi-Jian Yu, Lu Zeng, Xiang-Qian Luo, Xiao-Rui Geng, Rui Xu, Kun Chen, Gui Yang, Xi Luo, Zhi-Qiang Liu, Zhi-Gang Liu, Da-Bo Liu, Ping-Chang Yang, Hua-Bin Li

**Affiliations:** 1 Department of Otolaryngology, Affiliated Guangzhou Women and Children’s Medical Center, Guangzhou Medical University, Guangzhou, 510115 China; 2 Allergy Center, Otorhinolaryngology Hospital, the First Affiliated Hospital of Sun Yat-sen University, Guangzhou, 510080 China; 3 Center of Allergy & Immunology, Shenzhen University School of Medicine, Shenzhen, 518060 China; 4 Shenzhen ENT Institute, Longgang ENT Hospital, Shenzhen, 518116 China; 5 Department of Otolaryngology, Head and Neck Surgery, Affiliated Eye, Ear, Nose and Throat Hospital, Fudan University, Shanghai, 200031 China

## Abstract

It is recognized that T helper 2 (Th2) polarization plays a critical role in a large number of immune disorders. Yet, the remedies for reconciling the established Th2 polarization are still limited currently. Published data indicate that micro RNA-17-92 cluster is associated with the skewed immune response; 25 vitamin D3 (VD3) can regulate multiple bioactivities in the body. This study tests a hypothesis that VD3 facilitates the effect of specific immunotherapy (SIT) on Th2 response. We observed that treatment with either SIT or VD3 alleviated AR symptoms as well as reduced serum levels of specific IgE and T helper (Th) 2 cytokines, suppressed miR-19a (one of the members of the miR-17-92 cluster) and increased IL-10 in peripheral B cells, which was further improved in those AR patients treated with both SIT and VD3. The expression of miR-19a and IL-10 was significantly negatively correlated with each other in peripheral B cells of AR patients. Metabolites of VD3 formed a complex with retinoid acid receptor to repress the expression of miR-19a in B cells. We conclude that administration with VD3 promotes the effect of SIT on suppression of AR via repressing the expression of miR-19a in peripheral B cells.

## Introduction

In some predisposed individuals, harmless foreign substances, such as inhaled particles, airborne pollens, may trigger aberrant immune responses and cause immune inflammations in the local tissue such as airway allergy. Published data indicate that the incidence of allergic rhinitis (AR) is rising rapidly in the recent decades^1^. The pathogenesis of AR is to be further investigated. Current therapies for AR are not satisfactory in fact. Thus, it is important to further elucidate the pathogenesis of AR. To develop novel and effective therapies for AR is of significance.

MicroRNA (miRNA) is a class of noncoding RNA molecules encoded by endogenous genes; the length is about 18–22 nucleotides. miRNAs are found in plants and animals involved in post-transcriptional regulation of gene expression. Most miRNA genes are present as a single copy, multi copies or gene cluster existing in the genome^2^. miRNAs may bind target genes to break the genes or inhibit target gene transcription^3^. One of the best characterized miRNA clusters is the human miR-17-92 cluster, also known as oncomiR-1, encodes six hairpin transcripts carrying six miRNAs (miR-17, miR-18a, miR-19a, miR-20a, miR-19b-1, and miR-92a), located on human chromosome 13, within the third intron of the primary transcript C13orf25^4^. Elevated expression of miR-17-92 has been observed in a variety of immune disorders, such as cancer^5^ and allergic asthma^6^. The regulation of the miR-17-92 cluster has not been fully understood yet.

It is reported that vitamin D3-deficiency is associated with the pathogenesis of allergic disorders, such as asthma^7^ and AR^8^. Administration of 25 vitamin D3 (VD3, in short) can exert anti-inflammatory effects via regulating the biosynthesis of pro-inflammatory molecules in the prostaglandin pathway or through nuclear factor kappa-B by affecting cytokine production and inflammatory responses^9^. Whether the regulation of the miR-17-92 cluster is involved in the effect of VD3 on suppression of immune inflammation has not been investigated.

Specific immunotherapy (SIT), also known as “desensitization” or “hypo-sensitization”, is a medical therapy for allergic diseases^10^. The effects of SIT include attenuation of allergic symptoms, generation of tolerant immune cells, induction of blocking antibodies such as IgG4^11^. SIT has been used in the treatment of allergic diseases for many years. The therapeutic effect has been recognized, but needs to be further improved^12^. We and others found that combination of SIT and other remedies, such as using probiotics, promoted the effect of SIT^13, 14^. Based on the information above, we hypothesize that the therapeutic effect of SIT can be strengthened by VD3 via regulating the expression of a miR-17-92 cluster. In this study, we found that the expression of miR-19a, one of the miR-17-92 cluster members, was higher in the peripheral B cells in AR patients. The addition of VD3 significantly enhanced the therapeutic effect of SIT on AR symptoms.

## Results

### VD3 facilitates SIT in allergic rhinitis (AR)

**Figure 1:**
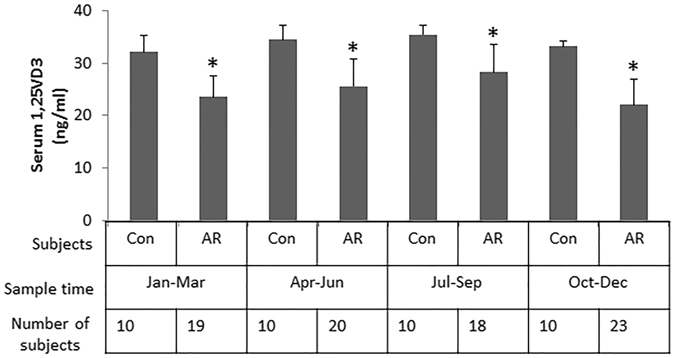
Assessment of serum 25VD3. The bars indicate the serum levels of 25VD3 in healthy subjects (Con) and AR patients, which were recruited in different seasons. *p < 0.01, compared with the control group.

**Figure 2:**
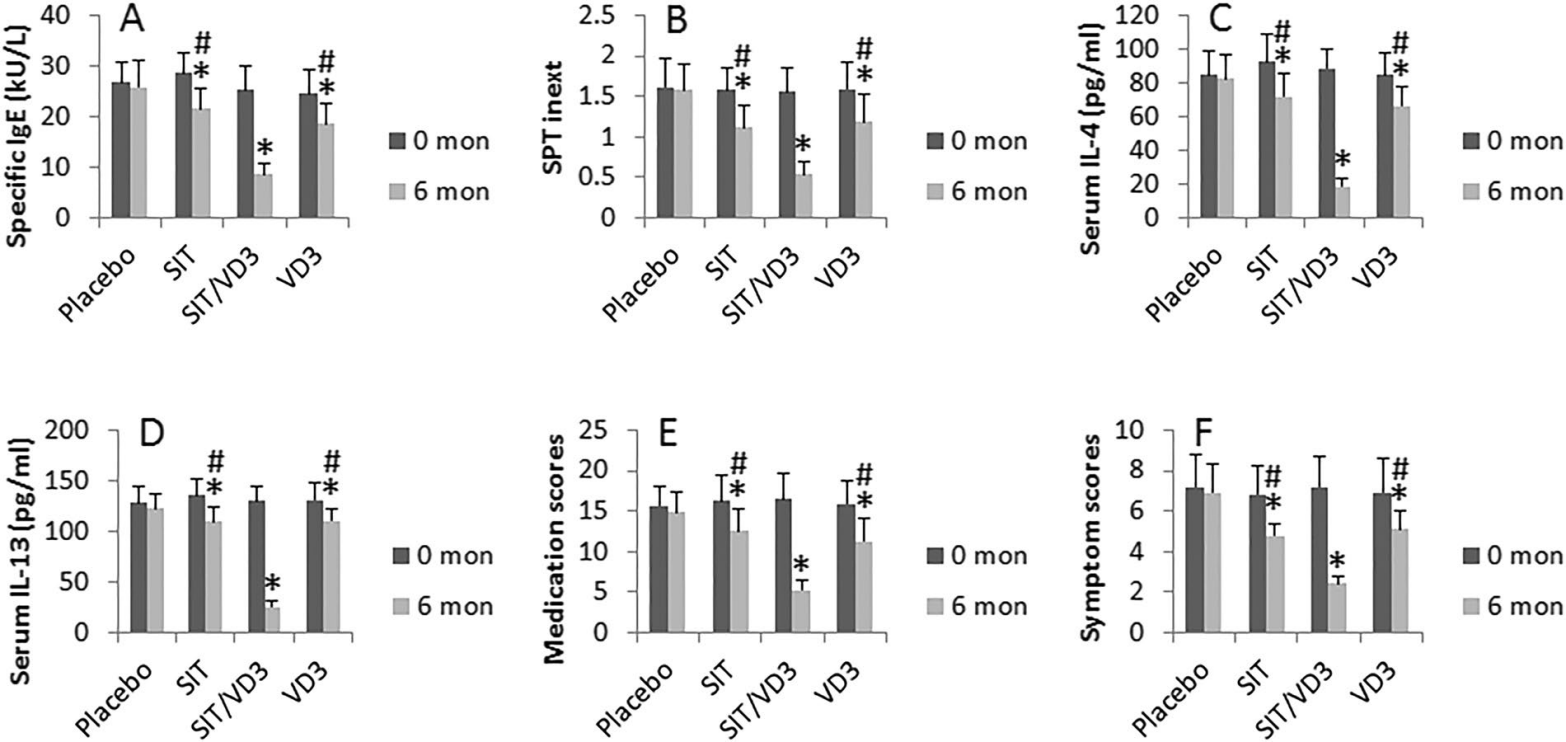
VD3 facilitates SIT in AR. The bar graphs show the AR-related parameters collected at 0 month (mon) and 6 months from the beginning of treatment. The treatment of AR patients (20 patients/group) is denoted on the X axis. Data of bars are presented as mean ± SD. *p < 0.05, compared with the placebo group. ^#^p < 0.05, compared with the SIT/VD3 group.

We firstly assessed the VD3 levels in the sera of AR patients. The results showed that although the VD3 levels were different from seasons to seasons, the serum VD3 levels were overall lower than that in healthy controls (Fig. 1). AR patients were randomly divided into 4 groups and treated with SIT or VD3 or SIT/VD3 or placebo, respectively. The therapeutic effect was reviewed 6 months from the beginning of the treatment. As shown by Fig. 2, the AR-related parameters, including serum specific IgE, SPT index, serum levels of Th2 cytokines (IL-4 and IL-13), total AR symptom scores and medication scores, were significantly down regulated in the SIT/VD3 groups as compared to the parameters before treatment. Treatment with SIT alone or VD3 alone also down regulated the AR-related parameters, but much less effective as compared with the SIT/VD3 group. The placebo group did not show detectable improvement of AR symptoms.

### VD3 facilitates the effect of SIT on suppression of miR-17-19 cluster in peripheral B cells of AR patients

**Figure 3:**
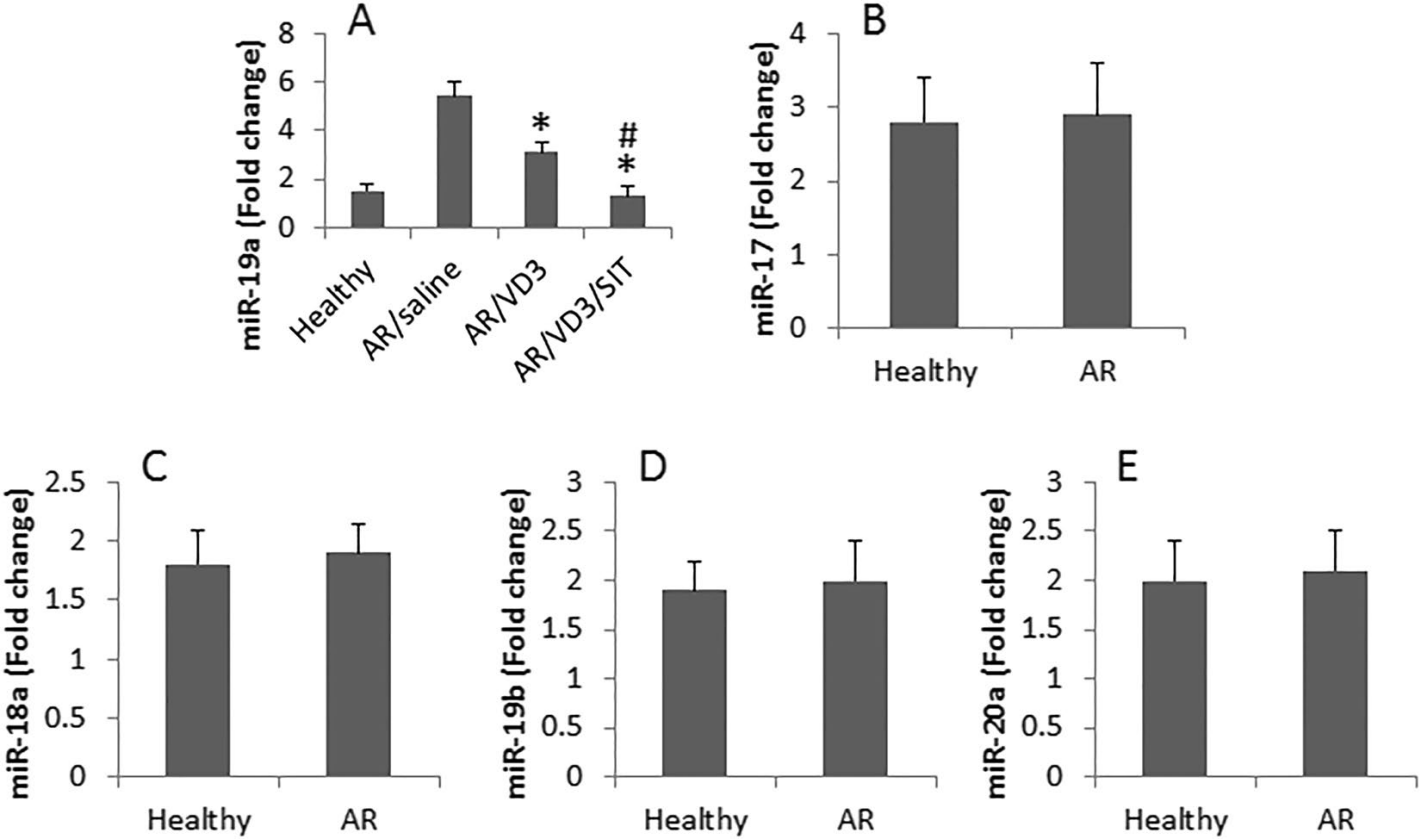
Assessment of miR-17-92 in peripheral B cells. B cells were isolated from peripheral blood samples of patients with AR (20 patients/group) and healthy subjects (n = 20). The cells were analyzed by RT-qPCR to assess the expression of miR-17**-**92. The bars (mean ± SD) indicate the miRNA levels in peripheral B cells in AR patients and healthy subjects. *p < 0.01, compared with healthy subjects. ^#^p < 0.01, compared with AR/VD3 group.

To investigate the mechanism by which administration with VD3 facilitates the therapeutic efficacy of SIT, we evaluated the regulatory effect of VD3 on the expression of a miR-17-92 cluster in peripheral B cells in AR patients based on published data that miR-17-92 cluster plays an important role in promoting Th2 polarization^6^. Peripheral blood samples from AR patients and healthy subjects were collected before and 6 months from the beginning of the treatment. B cells were isolated from the samples and analyzed by RT-qPCR. The results showed that only miR-19a, but not miR-19b, miR-17, miR-18a, or miR-20a, was higher in B cells from AR patients than that of healthy subjects. Treatment with VD3 markedly suppressed the levels of miR-19a in B cells (Fig. 3). These data demonstrate that miR-19a is specifically elevated in peripheral B cells in AR. The data further demonstrated that treatment with SIT or VD3 down regulated the expression of miR-19a in B cells, which was further down regulated by treatment with both SIT and VD3 (Fig. 3A).

### miR-19a mediates IL-4-suppressed IL-10 expression in B cells

**Figure 4:**
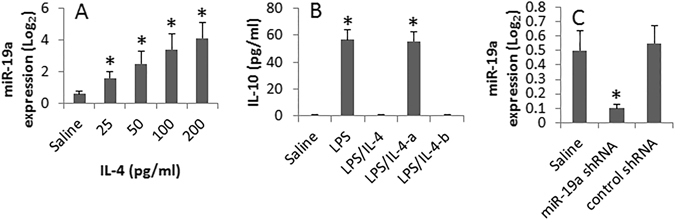
miR-19a mediates IL-4-suppressed expression of IL-10 in B cells. B cells were isolated from the peripheral blood samples of healthy subjects. (**A**) The cells were stimulated with IL-4 (the doses are denoted on the X axis) overnight. The bars (mean ± SD) indicate the miR-19a levels (by RT-qPCR). (**B**) Wild and miR-19a-deficient B cells were stimulated with LPS (10 µg/ml) or LPS/IL-4 (200 ng/ml) for 48 h in the culture. The bars indicate the levels of IL-10 in B cell culture supernatant (**B**; by ELISA). (**A**) miR-19a-deficient B cells. (**B**) B cells were treated with control shRNA. (**C**) The bars show the RNAi results of miR-19a in B cells (**C**; by RT-qPCR). *p < 0.01, compared with the saline group. The data are representatives of 3 independent experiments.

We next assessed the role of IL-4, the signature cytokine of Th2 response, in the regulation of miR-19a in B cells. We isolated peripheral B cells from healthy subjects. The B cells were stimulated by IL-4 in the culture for 48 h and then analyzed by RT-qPCR. The results showed that exposure to IL-4 did increase the expression of miR-19a in B cells in an IL-4 dose-dependent manner (Fig. 4A). We then stimulated B cells with LPS in the culture with or without the presence of IL-4. IL-10 levels in the culture supernatant were evaluated by ELISA. The results showed that exposure to LPS increased the levels of IL-10 in the culture, which were suppressed by the presence of IL-4. The data were corroborated by knocking down the miR-19a experiments (Fig. 4B,C).

### miR-19a is negatively correlated with IL-10 expression in peripheral B cells of AR patients

**Figure 5:**
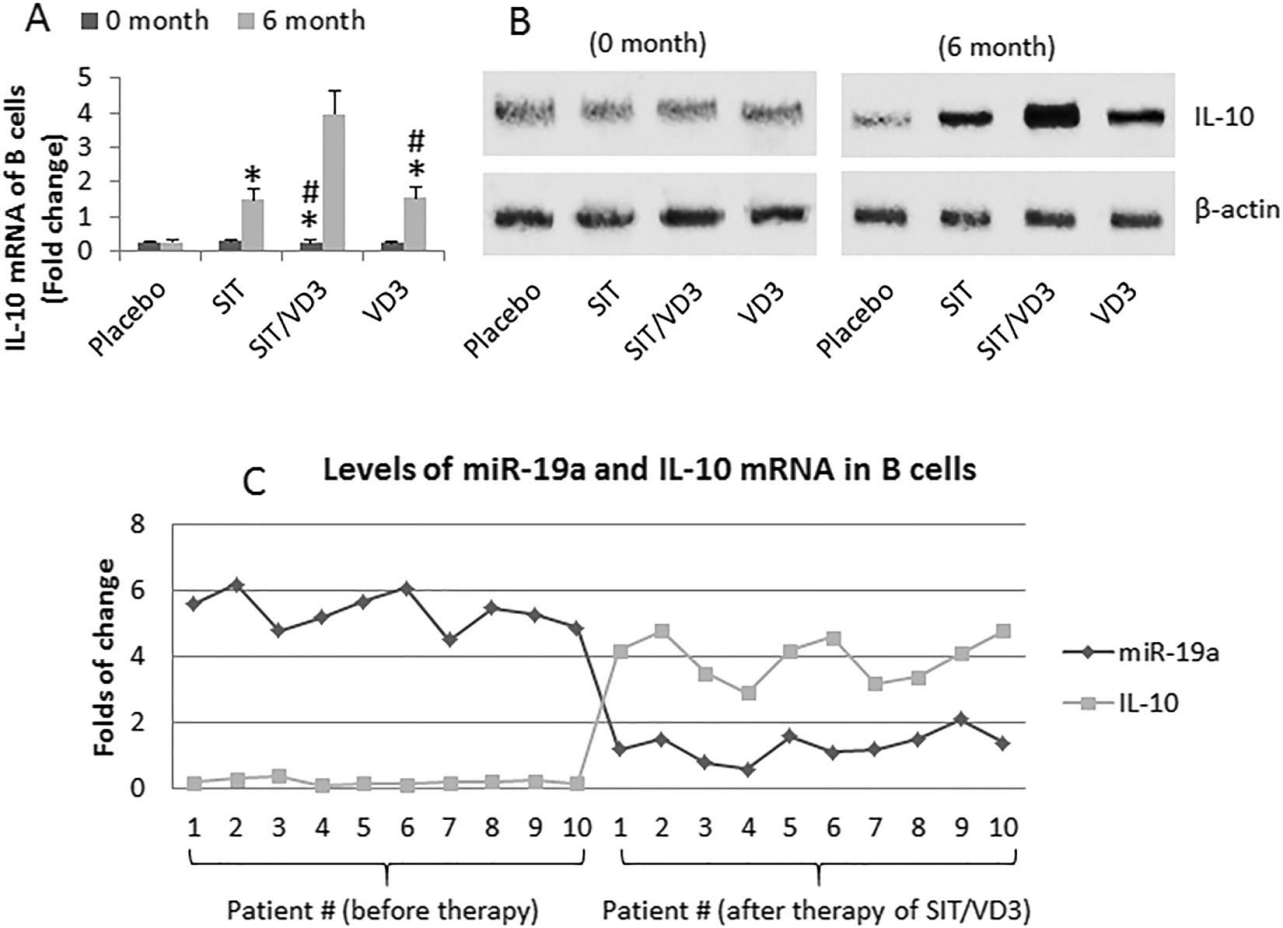
IL-10 expression in B cells and its correlation with miR-19a. (**A**,**B**) (20 patients/group), peripheral B cells of AR patients were prepared before the therapy (0 month) and 6 month after the start of the therapy (as denoted on the X axis); the B cells were analyzed by RT-qPCR and Western blotting. (**A**) The bars indicate the IL-10 mRNA in B cells (mean ± SD). (**B**) The immune blots indicate the protein levels of IL-10. (**C**) The curves indicate the individual data of miR-19a levels and IL-10 mRNA levels in the B cells of 10 randomly selected AR patients. *p < 0.01, compared to the placebo group. ^#^p < 0.01, compared with the SIT/VD3 group. The full-length gels and blots are included in the supplementary information.

Published data indicate that the production of IL-10 by B cells plays an important role in SIT^13^. We inferred that VD3 might be also associated with the regulation of IL-10 expression in B cells. To test this, we assessed the expression of IL-10 in peripheral B cells in AR patients before and after treatment with SIT or/and VD3. As shown by RT-qPCR, the expression of IL-10 was not altered in the peripheral B cells of AR patients treated with placebo. Treatment with either SIT or VD3 increased the expression of IL-10 in B cells, which was further increased by treatment with both SIT and VD3 (Fig. 5). To elucidate if the expression of miR-19a was correlated with the expression of IL-10 in B cells of AR patients, we performed a correlation assay with the expression of miR-19a and IL-10 in B cells of randomly selected 10 AR patients. The results showed a significant negative correlation (r = −0.92323; p < 0.01) between miR-19a and IL-10 mRNA in B cells.

### VD3 represses miR-19a gene transcription in B cells

**Figure 6:**
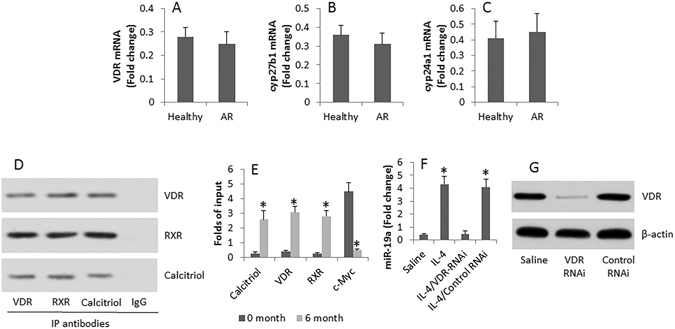
VD3 modulates miR-19a transcription in B cells. (**A**–**C**) The bars indicate the expression of VDR (**A**), cyp27b1 (**B**) and cyp24a1 (**C**) in peripheral B cells of healthy persons and AR patients. (**D**) The immune blots indicate a complex of VDR, RXR and calcitriol in peripheral B cells of AR patients at month 6 after the commerce of the therapy of SIT/VD3. (**E**) The bars indicate the levels of calcitriol, VDR, RXR and c-Myc at the miR-19a promoter locus. (**F**) The bars indicate the miR-19a levels in B cells after the treatment denoted on the X axis. (**G**) The immune blots show the VDR RNAi results in B cells. The data are representative of 3 independent experiments. Data of bars are presented as mean ± SD. *p < 0.01, compared with “0” month (**E**) or saline group (**F**). The full-length gels and blots are included in the supplementary information.

We then assessed the levels of VDR, cyp27b1 and cyp24a1 in peripheral B cells. The results showed that the mRNAs of VDR, cyp27b1 and cyp24a1 were detected in peripheral B cells of both healthy subjects and AR patients. Although there is no significant difference between healthy subjects and AR patients, the data indicate that peripheral B cells are capable of metabolize VD3 and respond to VD3. Observed the role of VD3 in regulating miR-19a gene transcription in B cells. As aforementioned, B cells were collected from the AR patients at 0 month and 6 month respectively. The cell extracts were analyzed by immunoprecipitation assay. The results showed a complex of calcitriol (the metabolites of VD3)/VD receptor (VDR)/retinoid acid X receptor (RXR) (CVR) in the B cells collected at month 6 (Fig. 6A). Further analysis showed that the CVR complex components at the miR-19a promoter locus, which significantly suppressed the binding of c-Myc to the promoter (Fig. 6B). The results implicate that the CVR complex binds miR-19a promoter to interfere with the gene transcription of miR-19a. To test this, we stimulated B cells with IL-4 in the culture; it resulted in miR-19a expression in the B cells (Fig. 6C), which was abolished by knocking down the VDR gene in the B cells (Fig. 6C,D).

## Discussion

The present data indicate that the expression of miR-19a was higher in the peripheral B cells of AR patients than that in healthy subjects. Treating AR patients with VD3 markedly increased the effect of SIT on suppression of AR symptoms via regulating the expression of miR-19a and IL-10 in peripheral B cells. The data suggest that concurrent administration of SIT and VD3 can be a novel remedy for AR.

The miR-17-92 cluster has several members. The present data show that miR-19a, but not the rest members of the miR-17-92 cluster, was significantly higher in peripheral B cells of AR patients than that in healthy subjects. The data demonstrate that the members of the miR-17-92 cluster can be individually regulated in B cells. Other investigators also found similar phenomenon. Simpson *et al*. indicate that miR-19a upregulates in asthma airway T cells to promote Th2 cytokine production^6^. Haj-Salem *et al*. reported that miR-19a facilitated the airway epithelial cell proliferation to contribute to the pathogenesis of airway allergy^15^. The data suggest that miR-19a may be a therapeutic target for the treatment of allergic diseases.

VD3 has been used in clinic as a supplement of VD. It is also used to treat some immune disorders. Such as Zhang *et al*. indicate that VD3 regulates the development of chronic colitis by modulating both Th1 and Th17 activation^16^. Rajanadh *et al*. found that supplementation of VD3 is effective in improving the quality of life as well as clinical symptoms in asthma patients^17^. Our data are in line with these studies by showing that administration of VD3 significantly strengthened the effect of SIT on alleviating AR symptoms. Such an effect may be associated with the suppression of miR-19a in peripheral B cells.

The data also show that administration of VD3 and SIT increased the expression of IL-10 in peripheral B cells. The IL-10^+^ B cells are a fraction of regulatory immune cells since they suppress other immune cell activities^18^. In our previous studies, we found that significantly lower frequency of IL-10^+^ B cells was observed in mice with food allergy. After increasing the frequency of IL-10^+^ B cells, the food allergy pathological changes in the mice were improved^19^. Lee *et al*. treated food allergy patients with SIT and found peripheral IL-10^+^ B cells were increased. The authors explained that interferon-γ played a critical role in the increase in the frequency of IL-10^+^ B cells in the peripheral system^20^. Liao *et al*. observed that administration of Clostridium butyrate also up regulated the frequency of peripheral IL-10^+^ B cells in asthma patients^13^. The above information indicates that multiple factors can up regulate the frequency of peripheral IL-10^+^ B cells.

VD3 can be hydroxylated by its hydroxylase, cyp27b1, to become calcitriol; the latter can bind VD receptor and forms a complex with RXR (CVR) on the nuclear membrane. CVR moves to target genes to regulate the gene transcription^21^. Our data are in line with the previous studies by showing that a complex of CVR in peripheral B cells after treatment with VD3. Such a complex bound to the miR-19a promoter locus and repressed miR-19a gene transcription.

In summary, the present data show that high levels of miR-19a were detected in peripheral B cells of AR patients. Administration with VD3 significantly enhanced the effect of SIT on AR symptoms via suppression of miR-19a in B cells.

## Materials and Methods

### Patients

Patients with perennial allergic rhinitis (AR) were recruited into this study. The demographic data of the patients are presented in Table 1. The diagnosis of AR was based on AR history, positive dust mite allergen skin test and serum specific IgE levels (UniCAP®, Phadia, Sweden) were ≥0.35 kU/L. Exclusion criteria included the presence of acute or chronic infectious disorders, autoimmune diseases, malignancies or other inflammatory disorders that are contraindications to specific immunotherapy (SIT). The study was approved by the Committees of Medical ethics at Shenzhen University, Guangzhou Medical University and Sun Yat-sen University. All the methods involving humans were carried out in accordance with the approved guidelines. Written informed consent was obtained from all participants.

**Table 1:** Characteristics of AR patients.

Number of Subjects	80
Age (yr) (median)	34.6
Gender (Male/Female)	40/40
SPT (wheal diameter)*	
<3 mm	0
10**–**15 mm	51
>15 mm	29
HDM sIgE^#^	
17.5**–**50 KU/L	11
50**–**100 KU/L	66
>100 KU/L	3
With asthma	6
With conjunctivitis	3

*A wheal diameter greater than 3 mm of the negative saline control was considered SPT positive.

^#^The serum sIgE to HDM (*Der p* and *Der f*) was measured by the ImmunoCap test and a value of more than 0.35 kUA/l was considered a positive response.

### Skin prick test (SPT)

SPT was performed in all subjects with the following allergen extracts (ALK-Abelló, Hørsholm, Denmark): *D. pteronyssinus, D. farinae*, grass pollen mix, cat and dog dander, American cockroach, mould mix, tree pollen mix and weed pollens. The mean of the largest diameter of the wheal was recorded as the response to the SPT. Histamine (0.01 mg/ml) was used as a positive control and saline was used as a negative control. The skin test index was the ratio between the response to an allergen and the response to histamine.

### Blood sample collection and immune cell isolation

Peripheral blood samples were collected from each subject (20 ml/person/time) via ulnar vein puncture. The peripheral blood mononuclear cells (PBMC) were isolated by the gradient density centrifugation. CD19^+^ B cells were isolated from the PBMCs by the magnetic cell sorting (MACS) with commercial reagent kits (Miltenyi Biotech) following the manufacturer’s instructions. The cell purity was checked by flow cytometry. If the purity did not reach 95%, the MACS was performed again.

### Cell culture

The isolated B cells were cultured in RPMI1640 media supplemented with 10% fetal bovine serum (BSA), 100 U/ml penicillin, 0.1 mg/ml streptomycin and 2 mM L-glutamine. The cell viability was assessed by Trypan blue exclusion assay.

### SIT procedures and administration of VD3

The SIT was performed in AR patients using the house dust mite extracts (Allergopharma Joachim Ganzer KG; Reinbek, Germany), or saline (placebo), via subcutaneous injection in 1 ml saline. The patients were coded. The observers were not aware of the code to avoid the observer bias. The SIT was initiated at a dosage of 20 U, and was continued weekly with an increase in the dosage each week; the dosages were 20, 40, 80, 200, 400, 800, 2,000, 4,000, 8,000, 10,000, 20,000, 40,000, 60,000, 80,000 and 100,000 U, respectively. The maintenance dose was 100,000 U once a week until the end of the 6^th^ month. The VD3 was prescribed by physicians; the patients took one capsule (2000 IU) or a similar capsule containing placebo vehicle (Dongju Pharmaceutics, Guangzhou, China) daily. The serum VD levels were monitored monthly. None of the patients showed serum VD levels higher than 50 ng/ml during the treatment period.

### Nasal symptom score (NSS)

The severity of the nasal symptoms of the AR patients before and after treatment was assessed according to published procedures^22^. The global discomfort caused by AR was rated on a 0–10 scale, 0 being no symptom, and 10 being the maximal severity of the symptom. The NSS was recorded weekly.

### Medication scores

Following published procedures^23^ with modifications, the medication scores were recorded for each patient during the period of treatment. When necessary, the patients were allowed to take anti-histamine tablets (levocetirizine; 5 mg/tablet) or/and nasal spray of corticosteroid (mometasone). 1 point: used one nasal spray per day; 2 points: used one tablet per day; 3 points, used one spray and one tablet per day. The results are presented as the averages per week.

### Enzyme-linked immunosorbent assay (ELISA)

The serum levels of IL-4 and IL-13, and the levels of IL-10 in culture supernatant were assessed by ELISA with reagent kits (R&D Systems) following the manufacturer’s instructions.

### RNA extraction and quantitative RT-PCR (RT-qPCR)

Total RNA, including miRNA, was extracted from the isolated peripheral B cells using TRIzol Reagent (Invitrogen) according to manufacturer’s instructions. For miR-17-92 cluster detection, extracted RNA was reverse transcribed to cDNA using the PrimeScript™ RT reagent Kit (Invitrogen); the resulting cDNA was subjected to real-time PCR using SYBR Green ER qPCR Mix (Invitrogen). Reference gene RNA U6B (Invitrogen) was analyzed as an internal control. The Universal qPCR Primer was provided in the VILO kit and the forward primer for miR-17, 18a, 19a, 19b and 20a were purchasing from Qiagen. For mRNA detection, cDNA was synthesized using a reverse transcription reagent kit (Invitrogen). PCR was performed using SYBR Green Master Mix (Invitrogen). The results were normalized to the β-actin. The sequences of IL-10 primers for PCR are presented in Table 2.

**Table 2:** Primers used in the study.

Molecule	Forward	Reverse
miR-19a promoter	gcttacagtgcaggtagtga	ggagcacttagggcagtaga
IL-10	gttctttggggagccaacag	gctccctggtttctcttcct
VDR	tatgacctgtgaaggctgca	atcatctcccgcttcctctg
Cyp27b1	acctgacccacttcctgttc	tctgagtggagtgctgtctg
Cyp24a1	ctgtgatgaaagaggccacg	accatcatcctcccaaacgt

### Western blotting

Total proteins were extracted from B cells, fractioned by SDS-PAGE and transferred onto a PVDF membrane. After blocking with 5% skim milk for 30 min, the membrane was incubated with antibodies of interest overnight at 4 °C, and followed by incubating with the second antibodies (labeled with peroxidase) for 1 h at room temperature. Washing with TBST (Tris-buffered saline Tween 20) was performed after each time of incubation. The membrane was then developed with ECL (enhanced chemiluminescence) and photographed with an image station (UVI Image System, Cambridge, UK).

### Preparation of cytosolic and nuclear extracts

The isolated B cells were incubated with lysis buffer at 4 °C for 15 min, and centrifuged at 500× g for 10 min at 4 °C. The supernatant was collected as the cytosolic extract. The pellet was added with nuclear extract buffer and incubated for 15 min at 4 °C, followed by centrifugation at 13,000× g for 10 min at 4 °C. The supernatant was collected as the nuclear extract. The protein concentrations were determined by the Bradford method.

### Immunoprecipitation

Protein extracts were precleared with protein G-agarose beads for 2 h at 4 °C. The supernatant was incubated overnight at 4 °C with 2 μg of specific antibodies or isotype IgG (a negative control). The beads were collected by centrifugation. The immune complex on the beads was eluted by eluting buffer and subjected to Western blotting. The antibodies used in this part were purchased from Santa Cruz Biotech [VDR (D-6), RXR (D-20) and calcitriol (B1253M)].

### Chromatin immunoprecipitation assay (ChIP)

ChIP assay was performed with a reagent kit (Sigma Aldrich) following the manufacturer’s instructions. B cells were fixed with 1% formaldehyde for 15 min. The cells were then lysed and sonicated to shear the chromatin DNA. Cell lysate was precleared with protein G-agarose beads for 2 h at 4 °C. The supernatant was incubated overnight at 4 °C with 2 μg of specific antibodies or isotype IgG (a negative control). The precipitated antibody-chromatin complex was collected by incubation with protein G-agarose beads for 1 h at 4 °C. The beads were collected by centrifugation and then washed and eluted in elution buffer. DNA was recovered from the precipitated samples by reverse crosslinking at 65 °C for 4 h and digested with proteinase K for 1 h at 45 °C to remove proteins, then the immunoprecipitated DNA was recovered by phenol/chloroform extraction and ethanol precipitation. The DNA or input (10%, collected before antibody precipitation) was analyzed by qPCR with the miR-19a promoter primers presented in Table 2. The results were presented as folds of input.

### RNA interference (RNAi) of miR-19a

RNAi of miR-19a was performed on B cells with the shRNA reagents provided by GeneChem (Shanghai, China) following the manufacturer’s instructions. The sequences of miR-19a shRNA were AGTTGCACTACAAGAAGAATG. The RNAi effect on B cells was assessed by RT-qPCR.

### Statistics

Data are presented as mean ± SD. The difference between two groups was determined by Student t test or ANOVA if more than two groups. P < 0.05 was set as a significant criteria.
